# Eleven quick tips for writing a Bioconductor package

**DOI:** 10.1371/journal.pcbi.1012856

**Published:** 2025-03-19

**Authors:** Charlotte Soneson, Lori Shepherd, Marcel Ramos, Kevin Rue-Albrecht, Johannes Rainer, Hervé Pagès, Vincent J. Carey

**Affiliations:** 1 Friedrich Miescher Institute for Biomedical Research, Basel, Switzerland; 2 SIB Swiss Institute of Bioinformatics, Basel, Switzerland; 3 Department of Biostatistics and Bioinformatics, Roswell Park Comprehensive Cancer Center, Buffalo, New York, United States of America; 4 Department of Epidemiology and Biostatistics, City University of New York School of Public Health, New York, New York, United States of America; 5 MRC WIMM Centre for Computational Biology, MRC Weatherall Institute of Molecular Medicine, University of Oxford, Oxford, United Kingdom; 6 Institute for Biomedicine, Eurac Research, Bolzano, Italy; 7 Fred Hutch Cancer Center, Seattle, Washington, United States of America; 8 Channing Division of Network Medicine, Mass General Brigham, Harvard Medical School, Boston, Massachusetts, United States of America; CANADA

## Introduction

**Fig 1:**
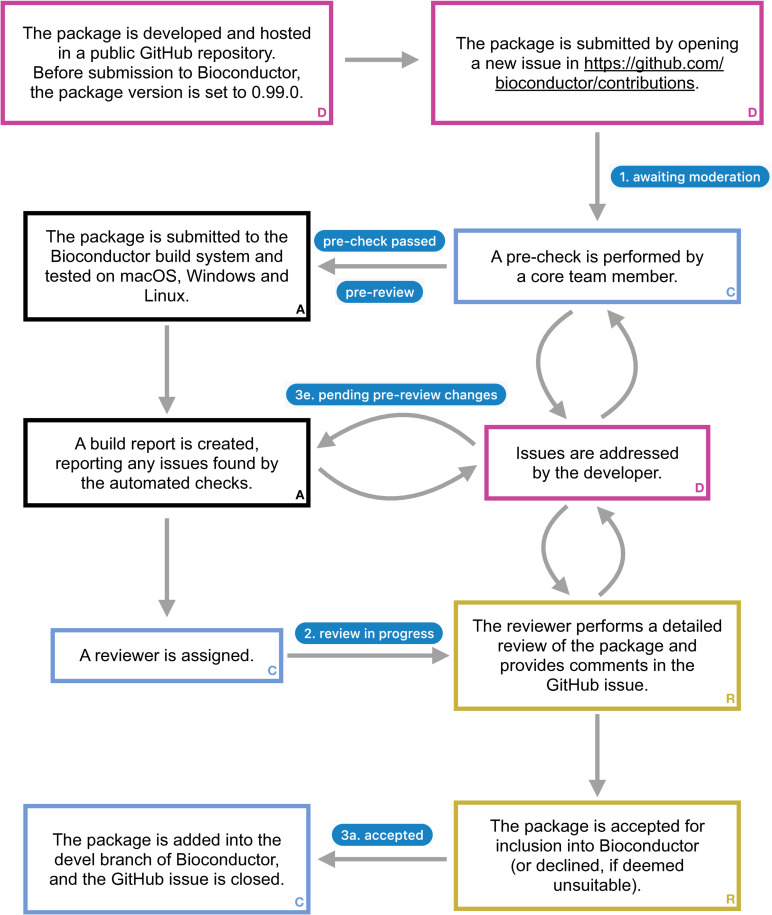
Overview of the Bioconductor package submission process. Pink boxes (with a **D** in the lower right corner) indicate steps performed by the package developer, blue boxes (**C**) are steps performed by a Bioconductor core team member, yellow boxes (**R**) are steps performed by the reviewer, and black boxes (**A**) represent automated steps performed on the Bioconductor build system. Blue badges correspond to labels added to the GitHub issue at the various steps. More details are available in the contribution guidelines [17]. The expected time from submission to acceptance depends on many factors, including the degree of adherence of the package to the Bioconductor guidelines, the comprehensiveness of the package’s code, as well as the overall load on the reviewer pool and the responsiveness of the package developer to address issues raised during review. In most cases, the process can be finalized within a few months.

Bioconductor [1–3] is a scientific software project aimed at developing and distributing open source software packages, mainly written in the R language, for analysis of biological data. Currently, (October 2024) more than 2,300 software packages are distributed via the project, in addition to over 900 annotation packages and more than 400 experiment data packages. The project employs a core team of developers [4], who among other things maintain essential hardware and software infrastructure. Most analysis packages; however, are not written by the core team, but developed by community members, and submitted for distribution via Bioconductor. Through submissions of code to Bioconductor, a large and diverse community of scientists, including experts on many different topics, share their methods and implementations with other developers and users. All packages submitted to Bioconductor are reviewed by a core team member or a trained community reviewer. The review takes place openly on GitHub [5], and allows for a constructive dialog between the author and the reviewer with the aim to improve the package and ensure that it fulfills defined quality standards and integrates well with existing Bioconductor packages. Fig 1 summarizes the package review process, from initial submission to acceptance into Bioconductor. After acceptance, the developer is expected to continue maintaining their package, which includes responding to bug reports, warnings and errors signaled by the Bioconductor build system, and usage queries on the Bioconductor support site, or, if necessary, to transfer the maintenance to another individual to avoid the package being labeled as ‘Deprecated’ and eventually removed [6]. This ongoing maintenance requirement ensures the long-term reliability and utility of Bioconductor packages for users and developers in their future work.

One characteristic feature of the Bioconductor ecosystem, which sets it apart from other R package outlets (e.g., CRAN [7] or R-universe [8]), is that many of the packages are tightly interconnected, and are intended and expected to work together in a concerted way. This allows a user to seamlessly assemble analytical workflows consisting of functions from many different packages, and enables reuse of robust, well-tested implementations of core functionality. All Bioconductor packages are tested jointly several times per week, to verify not only that the example code provided in the documentation of each package is indeed executable but also that all packages can be installed and function together. Twice yearly a new, joint release of the entire package ecosystem is created. One implication of this setup is that any new package that is added must adhere to a set of guidelines to enable a seamless interaction with existing packages. An extensive contributor’s guide is available to help developers navigate these guidelines [9]. In this Quick Tips article, we aim to provide accessible guidance for prospective contributors, focusing specifically on the aspects of the contribution guidelines that, in our experience, are most helpful to keep in mind throughout the development process in order to enable a smooth submission experience. In addition to these specific guidelines, there are many resources covering good software engineering practices [10–12], general R package development and generation of an initial package scaffold (e.g., devtools [13], R packages [14], usethis and biocthis [15,16]), which are equally valid also for Bioconductor packages and will not be repeated here. Similarly, while the tips provided here each represent an area where Bioconductor guidelines go beyond what is necessary for a minimal functional R package, they all correspond to good practices in R package development, which can be equally well applied to packages that are not aimed for submission to Bioconductor.

## Tip 1: Consider whether a new package is needed

Bioconductor distributes a large number of packages, suitable for analysis of many different types of data. Before embarking on building a new package, we strongly recommend searching the current set of packages to ensure that the intended functionality is not already available. A useful way of browsing existing packages is via the *biocViews*. These are mandatory keywords, chosen from a pre-defined collection, that indicate what type of functionality a package provides (e.g., packages related to gene expression analysis are annotated with the “GeneExpression” biocViews term [18]). Source code for all Bioconductor software packages is also browsable online [19]. If similar functionality is already available in an existing package, we suggest that the authors reach out to the maintainers of this package to investigate the possibilities of collaboration and consolidation of the contributions into a single package. Authors of a new package are encouraged to add a short paragraph in the documentation, positioning their package relative to other, related ones. This helps future users navigate the package ecosystem and find the right package for their needs, and highlights unique contributions of each package. Finally, in cases where the novel contributions are in the form of ‘wrapper code’ combining existing functions into a complete workflow for a specific task, these may be better submitted as a *workflow* package [20] rather than a new software package.

## Tip 2: Choose package and function names carefully

Conflicting package and function names often create confusion and considerably lower the usability of software, especially for new users. Therefore, the name of the package should be carefully selected, ideally early in the development process. Renaming of accepted Bioconductor packages is only rarely done, and effectively involves the deprecation of the old package and re-submission of the renamed one. For submission to Bioconductor, the package name cannot coincide with the name of a package that is already present on CRAN or Bioconductor. Ideally, to increase findability the name should also be descriptive of what the package does, and it should not be offensive. The Bioconductor contributor’s guide [21] provides more details related to package naming, and the available() function from the BiocManager R package [22] can be used to determine whether a package name is available. In selected cases, Bioconductor’s Code of Conduct Committee will be involved during the package review process to evaluate the appropriateness of a package name. In addition to the package itself, we also recommend thinking carefully about names of functions, arguments and variables within the code. Consistency of argument names across the functions in a package, and descriptive argument names, makes the use of the package by others much easier. Due to the interdependent nature of the Bioconductor ecosystem, package functionalities often share similar names. Thus, it is important that developers avoid collisions in function names where possible and learn about the roles of “generic” functions in the Bioconductor ecosystem. More details on these are given in Tip 4 below.

## Tip 3: Choose an approved open source license

All packages submitted to Bioconductor have to be released under an open source software license, to allow the community to use and build upon the wealth of code available within the project [23]. In particular, licenses restricting use (e.g., for commercial purposes) are not suitable for packages submitted to Bioconductor. Currently, the most popular licenses for Bioconductor packages are various flavors of GPL, the Artistic-2.0, and the MIT license [24].

## Tip 4: Use existing infrastructure and data representation formats

Bioconductor is built around a core set of infrastructure classes, generics, and methods for data representations. The contributor’s guide provides a list of the most common such classes [25]. For example, core packages provide classes to represent genomic sequences, genomic ranges and intervals, rectangular data (such as gene expression or protein abundance matrices), mass spectrometry data, images, and many other types of data. Each of these classes comes with a clear format specification as well as a set of validity checks and associated methods. Data analysis packages, in turn, provide additional functions that typically expect data in one of these standard formats as input, and return results in a (possibly different) standard format. The presence of these standard classes, and the expectation that they should be used by Bioconductor software packages, enables the construction of workflows involving multiple functions, from potentially many different Bioconductor packages, without the need to reformat the data or learn new data formats along the way. It also eliminates the need to reimplement the same functionality in several places—instead, a single, efficient, and well-tested implementation can be provided. For example, Bioconductor contains core functionality for reading and writing most common file formats used in bioinformatics. Use of the standard classes wherever applicable, or extending them to accommodate specific data representations, is a requirement for a package to be accepted into Bioconductor, and provides plenty of opportunity for interoperability with existing packages [25]. To avoid the need for extensive refactoring, we recommend thinking about the data representation early in the development process, and choosing a suitable format depending on the type of data.

## Tip 5: Develop your package against the appropriate R version

At any point in time, there exist two versions, or *branches*, of Bioconductor: *release* and *devel*. When a new package is accepted, it is added to the *devel* branch. This branch is where substantial development of packages is happening, and where new features are added. Due to this volatility, practical data analyses are typically not performed using the devel branch. Twice a year (in April/May and October/November), a new *release* version of Bioconductor is created from the current state of the devel branch. Development and addition of new features can then continue in the devel branch, while the release branch remains stable for the next 6 months, with the exception of critical bug fixes and minor adaptations. Historical release versions also remain available for reproducibility purposes.

As a developer, it is important to understand the concept of the release and devel branches, since it also affects the version of R that a package is tested with. Each new Bioconductor release is built for, and tested on, the most recent release version of R at the time of the Bioconductor release. For the spring Bioconductor release, which typically happens shortly after the yearly release of a new major R version, that means that in the development cycle leading up to the Bioconductor release, new packages should be developed and tested using what is then the development version of R (see [26] for information about the right version of R to use for the current release and devel version).

## Tip 6: Use consistent coding style

Using a consistent coding style simplifies both maintenance and code review. Well-structured code also makes contributions from other community members much easier (see also Tip 1). Bioconductor provides extensive guidelines on the desired coding style [25], and the BiocCheck package [27] will detect deviations from these guidelines and generate a helpful report pointing to the precise locations in the code that need attention. Several R packages for automatic styling and linting of R code exist (e.g., styler [28], lintr [29]), and some Bioconductor-adapted styles are available for these packages, in particular bioc_style() from the biocthis package [16], which can be directly used together with styler.

## Tip 7: Consult existing packages or the community for help and advice

If you run into problems or need assistance while developing a Bioconductor package, there is often help to be found within the community. The yearly Bioconductor conferences regularly feature instructor-led workshops on package development. The Slack workspace [30] has a large collection of channels dedicated to different topics, as well as a broader #developers-forum channel for general questions about package development and a #package-submission channel for general questions regarding the Bioconductor submission process. The slack workspace is also an excellent place for sharing experiences and helping others who may be struggling with similar issues. The bioc-devel mailing list [31] is another great way to reach out to the Bioconductor developer community for advice. Recently, a developer mentorship program provided opportunities for new developers to team up with an experienced developer and get personalized guidance during package writing [32], and the contributor’s guide [17] provides all the necessary details about the various steps in the process. Finally, the source code of all Bioconductor packages is available online [19]. This resource provides a rich source of inspiration and knowledge for new package developers, especially for more precise implementation-related questions.

## Tip 8: Include small example data and be mindful of the total package size

In order to showcase the functionality implemented in a package, small but realistic example data is almost always needed. There are several ways of accessing such data. Bioconductor contains a large number of *data packages*, many of which feature data that is useful for educational purposes [33,34]. If nothing suitable exists already, it is also possible to provide a small example data set within a software package, or to construct and submit a separate, but related, data package. If data is added directly in a software package, it is important to be mindful of limitations on the total package size [35], as well as to clearly document the original source of the data and how it was processed [36]. To build a separate data package, we strongly suggest using the ExperimentHub (for experiment data) [37] or AnnotationHub (for annotation data) [38]. Using the Bioconductor ‘Hubs’ allows larger data to be stored on trusted server locations, like zenodo [39], keeping the package itself lightweight. Data is downloaded only as needed and cached [40] when utilized. Creating an ExperimentHub package is also a great way to distribute data related to a publication and make it easily accessible to the community.

## Tip 9: Provide comprehensive, executable, and testable documentation

Good documentation is key to making a software package usable by the community. All Bioconductor packages are required to contain several levels of documentation. First, each user-visible (exported) function must have a documentation (or manual) page, describing all the function arguments and the expected output, as well as providing runnable examples of how to use the function. Second, the package also has to include one or more *vignettes*, which are long-form descriptive documentation, outlining how to use the package to perform a realistic analysis. These are excellent venues for showcasing the capabilities of a package, pointing to published reports and papers where the package was used in practice, and collecting answers to frequently asked questions. The vignettes must contain runnable code, and are tested several times per week to ensure that the provided code can indeed be executed without errors. The BiocStyle package [41] provides document formats that are recommended to use for Bioconductor vignettes.

## Tip 10: Add unit tests during the development process

Automated testing is essential for the robustness of a software package, and to ensure that all functionality is working as expected after an update of the package or any of its dependencies [10]. Targeted testing of individual functions and modules (‘unit testing’) is particularly useful for tracking down the precise source of potential bugs, and is strongly recommended for all Bioconductor packages. For example, separate unit tests can check that a function returns the expected error message if provided with the wrong type of input, and that it returns the expected value if appropriate input is supplied. R provides several frameworks for writing unit tests, including RUnit [42], tinytest [43], and testthat [44], which are all supported by Bioconductor. In most cases, it is easier and more convenient to write unit tests during, or even before, the development of a package, rather than at the end of the development process. Importantly, unit tests should test not only that the functions are running without errors but also that they return the expected results, for a variety of input data. This is another place where a set of small example data sets comes in handy, as the entire process of building and checking a package during the daily Bioconductor builds is limited to 40 min. If needed, special ‘long tests’ can be set up [45].

## Tip 11: Build and check your package regularly and before submitting

The first step that is taken after a new package is submitted to Bioconductor and deemed suitable in scope, is that the package is built and a series of automated checks are run (Fig 1). Typically, the review process for a package will not start until these automated checks pass without errors or warnings. Thus, we strongly recommend running similar checks for your package regularly during the development process, as well as just before submission, to detect any issues and be able to address them early on. General build and check tooling for R packages is provided via the R CMD build and R CMD check commands, or via the rcmdcheck R package [46]. Bioconductor-specific checks are performed by the BiocCheck package [27]. From a practical point of view, checks can either be run locally on the user’s machine, or be set up to run automatically using continuous integration platforms such as GitHub Actions, which makes it straightforward to test across multiple operating systems. The biocthis Bioconductor package [16], as well as the usethis [15] and rworkflows [47] CRAN packages, provide utilities to set up a continuous integration workflow from scratch for a package.

## Conclusions

In this article, we have provided a series of quick tips to assist R package developers interested in submitting their packages to Bioconductor. While these tips are useful for all Bioconductor packages, in some cases additional questions may come up, e.g., related to inclusion of Python, C++ or Fortran code, or development of R/Shiny applications. For this type of questions, we refer the reader to the comprehensive contributor’s guide [17], which provides a detailed overview of the guidelines that are currently in place.
